# Freely available, online videos to support neurological physiotherapists and students in task-specific training skill acquisition: a scoping review

**DOI:** 10.1186/s12909-024-05545-5

**Published:** 2024-05-31

**Authors:** Nicola C.M. Towersey, Kelvin Sasse, Verna Stavric, Gemma Alder, Nicola L. Saywell

**Affiliations:** School of Clinical Sciences, Department of Physiotherapy, Health and Rehabilitation Research Institute, Auckland University of Technology, Northshore Campus, Private Bag 92006, Auckland, 1142 New Zealand

**Keywords:** Task-specific training, Search engine, Neurological rehabilitation, Physiotherapy

## Abstract

**Background:**

Videos to support learning of clinical skills are effective; however, little is known about the scope and educational quality of the content of freely available online videos demonstrating task-specific training (TST). This review aimed to determine the extent, characteristics of freely available online videos, and whether the content is suitable to guide skill acquisition of task-specific training for neurological physiotherapists and students.

**Methods:**

A scoping review was conducted. Google video and YouTube were searched in December 2022. Videos that met our eligibility criteria and were explicitly designed for (TST) skill acquisition were included in the report.

**Results:**

Ten videos met the inclusion criteria and were difficult to find amongst the range of videos available. Most were presented by physiotherapists or occupational therapists, originated from the USA, featured stroke as the condition of the person being treated, and involved a range of interventions (upper limb, constraint induced movement therapy, balance, bicycling). Most videos were created by universities or private practices and only two used people with a neurological condition as the participant. When the content of videos and their presentation (instruction and/or demonstration), was assessed against each key component of TST (practice structure, specificity, repetition, modification, progression, feedback), five of the videos were rated very suitable and five moderately suitable to guide skill acquisition. Most videos failed to demonstrate and provide instruction on each key component of TST and were missing at least one component, with feedback most frequently omitted.

**Conclusions:**

There are many freely available online videos which could be described as demonstrating TST; very few are suitable to guide skill acquisition. The development of a standardised and validated assessment tool, that is easy to use and assesses the content of TST videos is required to support learners to critically evaluate the educational quality of video content. Guidelines based on sound teaching theory and practice are required to assist creators of online videos to provide suitable resources that meet the learning needs of neurological physiotherapists and students.

**Supplementary Information:**

The online version contains supplementary material available at 10.1186/s12909-024-05545-5.

## Background

Task-specific training (TST) is a common rehabilitation strategy used and taught by a wide range of allied health professionals including physiotherapists, occupational therapists, speech language therapists and academic institutions. It involves goal directed practice, repetition, progressive challenge, and positive reinforcement to optimise motor learning [1]. TST has been shown to be effective at inducing cortical reorganization, decreasing disability and improving functional outcomes for people with neurological conditions such as stroke, Parkinson’s disease, spinal cord injury and cerebral palsy [2–8]. TST is referred to in the literature by a range of terms, including ‘repetitive functional task practice’, ‘repetitive task practice’ [3], ‘task-orientated therapy’ [1] and ‘task-related training’ [9]. For this review, it will be referred to as task-specific training (TST).

The widespread use of the internet has extended traditional education by enabling users to search for, watch, and share a large variety of freely available online videos to supplement their learning. The use of online videos has grown in popularity over the past decade with ease of access and low cost making them one of the most frequently used self-learning resources for health professionals and students [10–12]. Videos have been shown to significantly improve learning outcomes [13], however, mechanisms for controlling the content within online videos are limited. The results of internet searches are determined by an algorithm using likes, views, and popularity rather than an assessment of whether the content is suitable to guide skill acquisition [14, 15] and previous studies have found online videos often omit key learning points and are of variable educational quality [14, 16–18].

Freely available online videos may be useful to guide skill acquisition and reinforce learning for neurological physiotherapists and students. However, there is a need to evaluate the quality of the content of these videos to ensure that they are based on best practice. To our knowledge, there has been no research exploring the extent, characteristics, and educational quality of freely available online videos for the skill acquisition of TST. In this scoping review, our aim was to determine the extent, characteristics, and whether the content of freely available online videos is suitable to guide skill acquisition of TST.

## Methods

A scoping review was conducted and carried out according to the Preferred Reporting Items for Systematic Review and meta-analysis for scoping reviews (PRISMA- ScR), adapting the five stages suggested by Arksey and O’Malley and the Joanna Briggs Institute (JBI) for evidence synthesis [21–23]. An a priori protocol was developed to guide this review prior to undertaking it.

### Identifying relevant videos

A detailed search plan outlining the search terms, sources, eligibility criteria, and delimiters was established based on suggestions by Godin et al. [24], and in consultation with a senior librarian. The JBI; Population, Concept and Context (PCC) elements were used to formulate a combination of search terms to maintain transparency, ensure organised search methods, and reduce the risk of bias [23]. The population included neurological physiotherapy, the condition of interest or intended audience, were left open. The concept was the demonstration and instruction of TST that a novice could use for skill acquisition. The context included rehabilitation. Refer to Table 1 for the initial search terms.

**Table 1:** Search terms

PCC elements	Search terms
Population	Physiotherapy Neurological physiotherapy “physical therapy” “neurological Physical therapy” neurological
Concept	“task oriented” “task specific” “task related” “motor relearning” “repetitive functional task”
Context	Rehabilitation Therapy Treatment Intervention training

The inclusion criteria included videos with the stated purpose to teach TST and included an element of physical skill demonstration and instruction. Videos had to be freely available online via a device with internet capabilities, in English, without subscription requirements to access, and within the scope of neurological physiotherapy practice [25]. There were no restrictions on the rehabilitation setting, date of upload, duration of video, or country of origin (Table 2).

**Table 2:** Eligibility Criteria

Inclusion criteria	Exclusion criteria
A video with an element of physical skill demonstration of task-specific training, using relevant physical or virtual objects to acquire/develop skills to perform everyday tasks with instruction on task-specific training.	Demonstration only
Stated purpose to teach task-specific training	Theory only
Available in English (includes subtitles)	
Within the scope of practice for neurological physiotherapists	
No monetary cost or requirement to register to gain access.	
Accessed through a device with internet capabilities (e.g., laptop, tablet, cell phone)	

A pilot search was carried out as suggested by the JBI guidelines [23] and following discussion between authors; search terms and the eligibility criteria were refined to reduce ambiguity regarding the definition of TST, and the level of instruction required for the video. For this review, TST included practice of meaningful tasks. Tasks were considered part-task if they were linked to a whole-task reconstruction; virtual reality involving the upper limb was considered part-task as it lacked the manipulation of physical objects and were included. Instructions needed to be provided during TST with sufficient detail to allow a novice to apply it. These could have been in the form of subtitles, voice-over, or directly to the camera.

Google video search engine and YouTube were searched due to their high use, free access and relevancy ordered results [26]. YouTube has 2,562 million active users, while Google is the dominant global search engine, with an 84.08% worldwide market share [26, 27]. Internet Protocol (IP) addresses and being logged into Google accounts have been found to contribute 11.7% to variation in results [19]. Therefore, searches were performed with personalised search off, in Google Chrome incognito browser, with relevancy sorting on, to improve the consistency of the search results. To reduce the effect of algorithmic searching, all preliminary searches were conducted on the Firefox search engine, with official searches performed in Google incognito, from the same IP address, on a single day in Auckland, New Zealand.

The searches were conducted from Auckland, New Zealand (NZ), on the 16th of December 2022 by KS. The complete search strategy used in Google video search engine and YouTube is in Appendix 1. Ten searches were conducted on each search engine with the first 10 pages of each search (representing 100 results) screened for relevance. This number was chosen to capture a wide range of the most relevant results, while still being feasible to screen [24]. Potentially relevant videos were bookmarked in a folder named after the search engine and in a subfolder with the search terms used [24]. If a video was embedded within a website, it was followed to its source and then bookmarked, to reduce duplicates.

Finally, a series of YouTube channels were hand searched to identify missed videos. Consistency was maintained by the same reviewer (KS), using the same IP address, applying the same method as the primary searches, on a single day (22nd December 2022).

### Video selection

Videos were selected using the following process. Duplicates were removed and KS screened all the videos against the eligibility criteria. A second reviewer (NT) independently cross-checked 20% of videos, to check for consistency and appropriate application of the selection criteria. Any discrepancies were referred to NLS to be resolved by consensus.

### Data charting

Data charting was used to synthesise and interpret the data [21]. KS and NT independently viewed the selected videos and summarised the data in a Microsoft (MS) Excel spreadsheet, based on the JBI guidelines [22, 23]. The data extracted for this review were the video title, upload date, duration, number of views, likes/ dislikes, the presenter(s), the participant(s), source, country of origin, intervention, the key components of TST (practice structure, specificity, repetition, modification, progression, feedback) and how they were presented (instruction and/or demonstration).

### Summarising and reporting results

To address the extent and characteristics of the selected videos, we identified trends using data analysing tools and pivot tables in MS Excel and presented the data descriptively.

### Scoring of videos

The suitability of videos to guide skill acquisition was assessed using a pragmatic scoring system developed for this scoping review. Although previous studies have developed scoring systems to evaluate online material [28, 29], none were appropriate for the purpose of this study. The scoring system was developed after reviewing pertinent literature [30, 31] and seeking expert opinion. It evaluated video content against the agreed key components of TST for motor learning (practice structure, specificity, repetition, modification, progression, feedback) and whether demonstration and instruction were provided on each component. Videos were scored from 0 to 2 for each of the six TST components for a total possible score of 12. Components scored 2 if both demonstration and instruction were provided, 1 if they provided either one, or 0 for a missing component. Those videos scoring 0–4 were considered unsuitable, 5–8 moderately suitable and 9–12 very suitable to guide skill acquisition. Data analysis was conducted by KS, and NT and NSW, physiotherapists with extensive clinical and teaching experience in neurological physiotherapy.

## Results

### Identification and selection of material

Google Video and YouTube searches resulted in 2,000 videos. After initial screening of titles and thumbnails, 179 were bookmarked as potentially relevant. Hand-searching YouTube channels identified an additional 12 videos, resulting in 191 videos being manually inputted into a MS Excel spreadsheet.

**Fig. 1:**
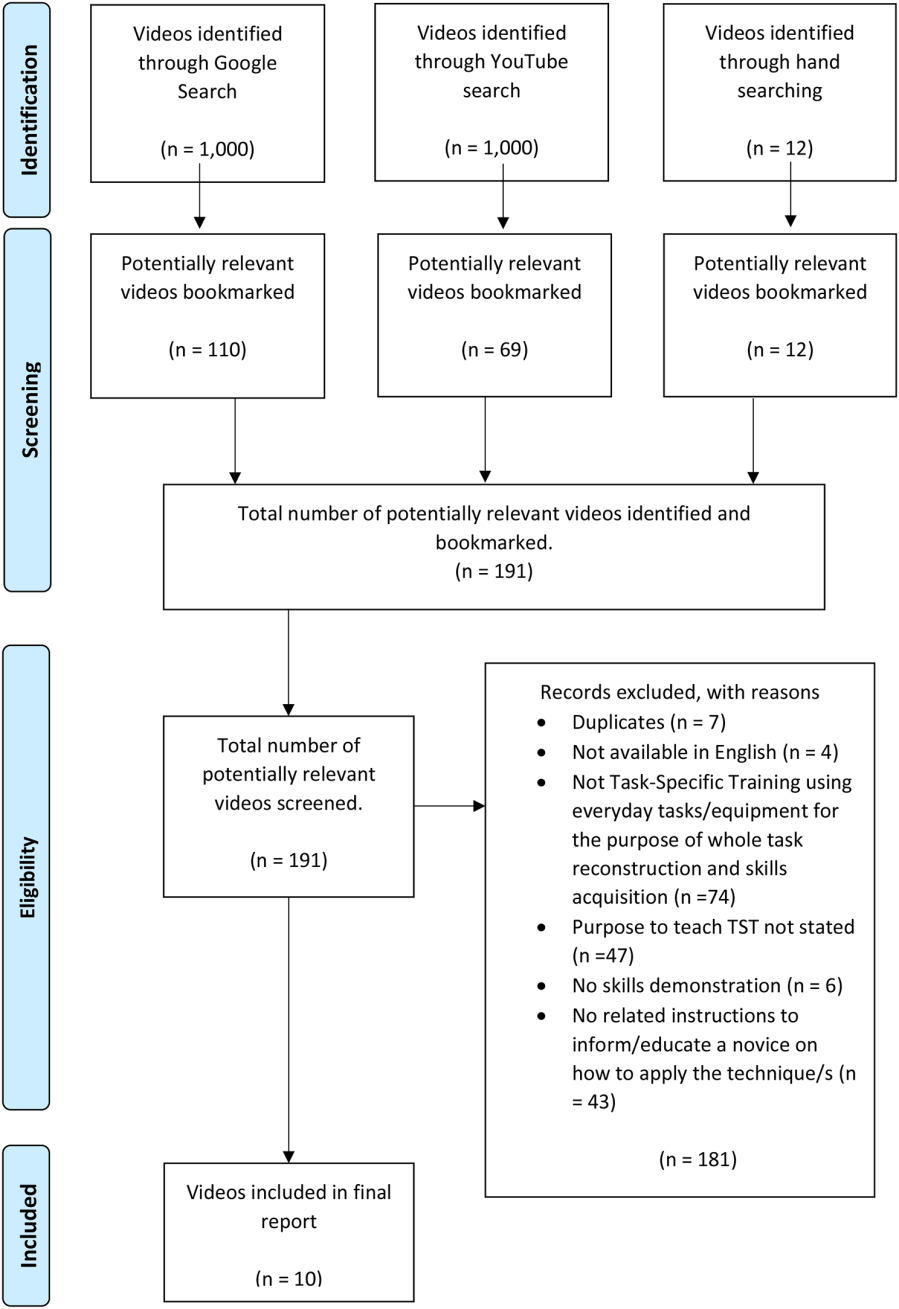
PRISMA flow diagram

All 191 videos were screened against the eligibility criteria by KS, with NT cross-checking 20% between January 11th, 2023, and January 13th, 2023. The agreement between reviewers was high (95%), exceeding the PRISMA-ScR guidelines requirements of 70–80% agreement [22]. A third reviewer (NLS) adjudicated any discrepancies (7%) with reference to the definition of TST and the inclusion criteria. Of the181 rejected videos, seven were duplicates, four were unavailable in English, 74 did not meet our definition for TST, 47 did not explicitly state that the purpose of the video was to teach TST, six had no skills demonstration in the video and 43 did not have sufficient instruction to allow a novice to repeat. Only ten videos fulfilled the eligibility criteria (0.4% of videos identified, 5% of videos bookmarked) and were included in the review, as represented in Fig. 1.

### Description of included videos

Videos had been uploaded to the platform a median of 30 months prior to our search date (range: 7–79 months). The median length of videos was 5.32 min (range: 3–20 min), with a median of 1386.5 views (range: 181–21950). The median number of likes for a video was 11.5 (range: 2–581), and the number of dislikes for all videos was 0. Occupational therapists were presenters in five of the videos, physiotherapists in two and the professional status of the presenter was unidentified in three videos. The focus of most videos was stroke rehabilitation (9) with cerebral palsy rehabilitation presented in one. Only two of the videos included people with the selected condition as participants. Seven videos were uploaded by universities, two by clinicians in a private practice and one by an unidentified contributor. Most videos were created in the United States of America (USA) with one each from Australia and India. A range of interventions were presented including upper limb training (7), bicycle training (1), constraint induced movement therapy (1), and balance training (1). A summary of the characteristics of the included videos is provided in Table 3.

**Table 3:** Characteristics of included videos

	Title/URL	Upload date	Duration	Views	Likes/dislikes	Presenter	Condition	Source	Country	Intervention
**1**	Upper Extremity Task Oriented Training Home Program for paralysis: Level 3	2-Apr-20	4:24	1,321	7/0	Unidentified	Stroke	Marquette University	USA	UL training
	https://www.youtube.com/watch?v=t715GdllWdM									
**2**	Upper Extremity Task Oriented Training Home Program for paralysis: Level 2	25-Mar-20	3:34	1,452	8/0	Occupational therapist	Stroke	Marquette University	USA	UL training
	https://www.youtube.com/watch?v=X1f8XoGiglA									
**3**	Hand over hand: A caregiver task oriented approach to stroke rehabilitation: A case report	25-May-16	20:28	1,828	16/0	Physiotherapist	Stroke (R)	Marymount University	USA	UL training
	https://www.youtube.com/watch?v=MfP70qmIziQ									
**4**	Task-specific training for bicycle-riding goals in ambulant children with CP	5-Oct-21	10:35	452	8/0	Physiotherapist	Cerebral palsy (R)	University of Melbourne	Australia	Bicycle training
	https://www.youtube.com/watch?v=BWA3mpaGZfg									
**5**	Upper Extremity Task Oriented Training Home Program for paralysis: Level 2	9-Apr-20	5:57	698	8/0	Occupational therapist	Stroke	Marquette University	USA	UL training
	https://www.youtube.com/watch?v=seRoasdKOrw									
**6**	Improve Hand Function After Stroke using Task-specific training	18-Dec-21	11:47	19,572	581/0	Occupational therapist	Stroke	Private practice	USA	UL training
	https://www.youtube.com/watch?v=uBi4z1hbbAQ									
**7**	Upper Extremity Task Oriented Training Home Program for paralysis: Level 1	18-Apr-20	3:26	2,1950	23/0	Unidentified	Stroke	Marquette University	USA	UL training
	https://www.youtube.com/watch?v=Jk3q73GfO8U									
**8**	How does Constraint-Induced Movement Therapy work / What is task-specific training	2-May-22	6:19	1,7861	62/0	Occupational therapist	Stroke	Private Practice	India	CIMT
	https://www.youtube.com/watch?v=s9upvAfNWSI									
**9**	Upper Extremity Task Oriented Training Home Program for paralysis: Level 1	29-Mar-20	4:28	885	15/0	Occupational therapist	Stroke	Marquette University	USA	UL training
	https://www.youtube.com/watch?v=Ty6lYQwlyko									
**10**	Task Specific Training	21-Apr-21	5:07	181	2/0	Unidentified	Stroke	Unidentified	USA	Trunk control, Balance
	https://www.youtube.com/watch?v=yvZ-_Z9LY0M									

(R) = People with the selected condition, as participants, USA = United States of America, UL = Upper limb, CIMT = Constraint induced movement therapy

Only 10 videos explicitly stated that the purpose of the video was to teach TST and therefore had their content assessed for its suitability to guide skill acquisition. The videos were assessed in relation to the key components of TST (practice structure, specificity, repetition, modification, progression, feedback) and how they were presented (with instruction and/or demonstration). All videos included demonstration and instruction about manipulating practice structure to promote motor learning. Specificity was demonstrated in all videos using physical objects relevant to the task, while only three videos provided instruction in addition to demonstration. Nine of the videos provided demonstration and instruction on the use of repetition, with one video providing instruction only. Five videos provided demonstration and instruction on how to modify the activity, three provided instructions only and two failed to provide modification. Seven videos provided demonstration and instruction on progressions, with two videos providing instruction only, and one failing to demonstrate or provide instruction on any form of progression. None of the videos demonstrated the provision of feedback to promote motor learning and only one video provided instruction on how to provide feedback. The scores for videos ranged from 6 to 9/12 with five videos being considered moderately suitable and five very suitable to guide skill acquisition. Only one video included all the key components of TST and none of the videos provided demonstration and instruction on each component. A breakdown of the TST components and suitability scores is detailed in Table 4.

**Table 4:** Task-specific training components and suitability scores

TST components	Video number																			
	1		2		3		4		5		6		7		8		9		10	
	D	I	D	I	D	I	D	I	D	I	D	I	D	I	D	I	D	I	D	I
Practice structure	✓	✓	✓	✓	✓	✓	✓	✓	✓	✓	✓	✓	✓	✓	✓	✓	✓	✓	✓	✓
Specificity	✓		✓		✓	✓	✓		✓		✓	✓	✓		✓		✓		✓	✓
Repetition	✓	✓	✓	✓	✓	✓	✓	✓	✓	✓		✓	✓	✓	✓	✓	✓	✓	✓	✓
Modification	✓	✓		✓				✓	✓	✓		✓	✓	✓	✓	✓	✓	✓		
Progression	✓	✓		✓	✓	✓	✓	✓	✓	✓		✓	✓	✓	✓	✓	✓	✓		
Feedback												✓								
**Total score /12**	9		7		8		8		9		8		9		9		9		6	
**Suitability**	**Very suitable**		Moderately suitable		Moderately suitable		Moderately suitable		**Very suitable**		Moderately suitable		**Very suitable**		**Very suitable**		**Very suitable**		Moderately suitable	

D = Demonstration, I = Instruction, Total score: 0–4 = Unsuitable; 5–8 = Moderately suitable = 9–12 = very suitable

## Discussion

This review was the first, to our knowledge, to examine the extent, characteristics, and suitability of freely available online videos that guide skill acquisition of TST for neurological physiotherapists and students. Despite a wide and comprehensive search strategy, only ten videos met the eligibility criteria. This suggests that despite over 2000 videos being available, there is a lack of suitable material to address the skill acquisition of TST for neurological physiotherapists and students.

This review highlights a fundamental problem when searching for educational videos online. Namely, it is difficult to find the few suitable videos amongst the array of videos of variable educational quality. It is unlikely that everyday internet users would be prepared to screen so many videos to find those ones related to TST which provide adequate information for training skill acquisition. Several authors have suggested mechanisms to improve the identification of educational videos. The use of a domain based ranking system, that ranks videos from trusted sources (universities or health organisations) higher up in the search results may make identification easier [32]. The use of inbuilt educational filters, with a strict criterion for labelling content as educational, might also improve identification [32, 33]. In addition, organisations with an interest in educating physiotherapists could identify and disseminate existing online videos that are suitable to guide skill acquisition.

During our review of the characteristics of videos, we noticed that physiotherapists and occupational therapists created the majority of TST videos using a range of interventions, mainly featuring the upper limb, and the condition of stroke. None of the videos demonstrated the use of TST during walking, which would be particularly useful for neurological physiotherapists and students, as this is often their focus in rehabilitation. People with a neurological condition used as participants were found in only two of the videos. One of the strengths of video is that it can depict authentic, real-world experiences of people with neurological conditions during rehabilitation sessions. The lack of involvement of people who have real impairments means subtleties in using TST skills for people with a neurological condition will be overlooked. Time constraints and ethical considerations may have been factors in the reduced involvement of people with neurological conditions, however, overcoming these issues to include people with real impairments would enhance the learning experience.

The suitability of videos to guide skill acquisition was assessed in relation to the key components of TST and how they were presented (with demonstration and/ or instructions) and were found to be variable. None of the videos provided demonstration and instruction for each component of TST, and only one video included all the key components. The use of instruction and demonstration has been found to optimise skill acquisition [34, 35], therefore, its omission would impact users’ understanding of the components and their ability to perform TST effectively. Feedback was omitted in all but one video, which was concerning as feedback is essential for motor learning and an integral component of TST [36, 37]. These results support the findings of previous studies, which found videos created for health professions to be of low educational quality and missing key information. Videos on surface anatomy omitted key aspects related to upper and lower limb anatomy, such as vessels, nerves, cubital fossa, wrists, and hands [28]. Videos by physiotherapists on shoulder joint mobilisation techniques failed to describe or illustrate vital aspects of techniques such as patient and therapist position, force of application and dosage [17]. Online videos have also frequently been found to contain inaccurate, contradictory, or misleading information [28, 32, 38, 39] with no reference to sources or evidence; this is in part due to the lack of peer review processes to monitor quality [38]. This highlights, that users need to critically evaluate the content of videos, and that improvements need to be made before they can be recommended as a valuable learning resource.

It has been suggested that the quality of video content for education can be filtered by evaluating variables such as the uploading source, video duration or the subjective estimation by viewers, expressed as likes/dislikes. The uploading source in particular has been found to be valuable for discriminating and predicting the quality of video content. Those uploaded by professionals, professional associations, and credible health care organisations are often of higher quality and are more suitable for education than those uploaded by individuals [38, 40–43]. In contrast, video duration and the likes/dislikes ratio were found to be unreliable as a predictor of quality [14, 40, 42, 43]. These results are consistent with the findings from our study which found that the videos sourced from educational institutions or private clinics were more suitable to guide skill acquisition. Neurological physiotherapists and students should consider the source of online videos to assist them to assess the educational quality of video resources.

This review highlights the importance of evaluating the content within freely available online videos. There are several tools available for assessing the quality, flow, and user friendliness of websites [44], evaluating health information on the internet, and the credibility of websites [45]. Although these tools are somewhat useful, they do not assess the content of the video in sufficient detail to help users determine if they are a suitable learning resource to guide skill acquisition. The development of content specific quality tools is required to assist learners to critically evaluate the quality of video content. In addition, guidelines based on sound teaching theory and practice are required to assist creators of online videos to create high quality resources that meet the needs of neurological physiotherapists and students.

### Strengths

This study was the first, to our knowledge, to examine the extent, characteristics of freely available online videos, and whether the content is suitable to guide skill acquisition of TST for neurological physiotherapists and students. Previous studies [38, 39] have evaluated the quality of online videos using various gross assessment tools [44–46]. However, these tools do not evaluate the video content; our study assessed whether the content of freely available online TST videos is suitable to guide skill acquisition. This review has identified difficulties neurological physiotherapists and students face in sourcing relevant videos of good educational quality without subscriptions to specialised domains. Attempts were made throughout the searches to reduce the impacts of search engine personalisation; and consistent reporting of the search strategy and methods, maintained rigour and transparency.

## Limitations

The criteria used to evaluate the suitability of the video content for skill acquisition was developed by the authors, was subjective, and may have been affected by observer bias. To reduce the risk of bias, two authors (KS, NT) assessed each video independently. The use of American spelling was used as it resulted in the most search results however, it may have influenced the identification of videos and resulted in the higher prevalence of videos from the USA. Forty-seven videos were excluded as they did not fit our inclusion criteria of explicitly stating that the purpose was the teach. It is acknowledged that some of these videos may have been videos teaching TST.

## Conclusions

There are very few suitable online videos that are freely available and specifically designed to support neurological physiotherapists and students in the skill acquisition of TST. The development of a standardised and validated assessment tool, that is easy to use and assesses the content of TST videos is required to support learners to critically evaluate the educational quality of video content. Guidelines based on sound teaching theory and practice are required to assist creators of online videos to provide suitable resources that meet the needs of neurological physiotherapists and students.

### Electronic supplementary material

Below is the link to the electronic supplementary material.

Supplementary Material 1

## Data Availability

The datasets used and/or analysed during the current study are available from the corresponding author on reasonable request.

## Abbreviations

TST: Task-specific training
USA: United States of America
CIMT: Constraint Induced Movement Therapy
UL: Upper limb
MS: Microsoft
JBI: Joanna Briggs Institute
NZ: New Zealand
IP: Internet protocol
PCC: Population, Concept, Context
