# Supplementary material for: Freely available, online videos to support neurological physiotherapists and students in task-specific training skill acquisition: a scoping review
Source: BMC Med Educ. 2024 May 31;24:603. doi: 10.1186/s12909-024-05545-5 (PMC11143672; doi:10.1186/s12909-024-05545-5)
Supplement: Supplementary file 1 — Supplementary Material 1 [file 12909_2024_5545_MOESM1_ESM.docx]

**Appendix 1:** Search strategy

Search results – Google Video Search

Google Video Search

Browser: Google chrome incognito, personalisation off, search by relevancy on.

The first 10 pages / 100 items were screened by title and thumbnail.

Date: 16^th^ December 2022.

Locations: Auckland, New Zealand

| # | Search Terms | # Results | Results screened | # new potentially relevant results |
| --- | --- | --- | --- | --- |
| 1 | “task oriented” neurological rehabilitation | 1340 | 100 | 37 |
| 2 | “task specific” neurological physiotherapy | 1619 | 100 | 27 |
| 3 | “task oriented training" | 1140 | 100 | 12 |
| 4 | “task related" neurological | 369 | 100 | 0 |
| 5 | motor relearning | 1510 | 100 | 6 |
| 6 | “repetitive functional task” neurological | 0 | 100 | 0 |
| 7 | repetitive functional task neurological | 26800 | 100 | 18 |
| 8 | "task specific" "physical therapist" neurological | 364 | 100 | 9 |
| 9 | "task oriented" "physical therapist" neurological | 411 | 100 | 1 |
| 10 | (“task oriented” OR “task specific OR “task related”) AND physiotherapy AND neurological AND rehabilitation | 1340 | 100 | 0 |
|  |  |  |  | **Total 110** |

Search results – YouTube

YouTube Search

Browser: Google chrome incognito, personalisation off, search by relevancy on.

The first 100 items were screened by title and thumbnail.

Date: 16^th^ December 2022.

Locations: Auckland, New Zealand

| # | Search Terms | Results | Results screened | # new potentially relevant results |
| --- | --- | --- | --- | --- |
| 1 | “task oriented” neurological rehabilitation | Unknown | 100 | 10 |
| 2 | “task specific” neurological rehabilitation | Unknown | 100 | 11 |
| 3 | “task specific” neurological physiotherapy | Unknown | 100 | 3 |
| 4 | “task oriented” physical therapy | Unknown | 100 | 6 |
| 5 | motor relearning | Unknown | 100 | 13 |
| 6 | repetitive functional task therapy | Unknown | 100 | 14 |
| 7 | task related training | Unknown | 100 | 0 |
| 8 | task specific training | Unknown | 100 | 2 |
| 9 | task oriented training | Unknown | 100 | 3 |
| 10 | (“task oriented” OR “task specific OR “task related”) AND (physiotherapy OR OT OR PT OR "physical therapy") AND neurological | Unknown | 100 | 7 |
|  |  |  |  | **Total 69** |
